# The Interaction of CD154 with the α5β1 Integrin Inhibits Fas-Induced T Cell Death

**DOI:** 10.1371/journal.pone.0158987

**Published:** 2016-07-08

**Authors:** Meriem Bachsais, Nadim Naddaf, Daniel Yacoub, Suzanne Salti, Nada Alaaeddine, Fawzi Aoudjit, Ghada S. Hassan, Walid Mourad

**Affiliations:** 1 Laboratoire d’Immunologie Cellulaire et Moléculaire, Centre Hospitalier de l’Université de Montréal, 900 rue Saint-Denis, Tour Viger, Room 10-482, Montréal, QC, Canada; 2 Department of Pathology, 11-5076, Faculty of Medicine, St Joseph University, Beirut, Lebanon; 3 Centre de recherche en immunologie et rhumatologie, CHUL, 2705, Boul Laurier, QC, Canada; Thomas Jefferson University, UNITED STATES

## Abstract

CD154, a critical regulator of the immune response, is usually associated with chronic inflammatory, autoimmune diseases as well as malignant disorders. In addition to its classical receptor CD40, CD154 is capable of binding other receptors, members of the integrin family, the αIIbβ3, αMβ2 and α5β1. Given the role attributed to integrins and particularly the β1 integrins in inhibiting apoptotic events in normal as well as malignant T cells, we were highly interested in investigating the role of the CD154/α5β1 interaction in promoting survival of malignant T cells contributing as such to tumor development and/or propagation. To support our hypothesis, we first show that soluble CD154 binds to the T-cell acute lymphoblastic leukemia cell line, Jurkat E6.1 in a α5β1-dependent manner. Binding of soluble CD154 to α5β1 integrin of Jurkat cells leads to the activation of key survival proteins, including the p38 and ERK1/2 mitogen-activated protein kinases (MAPKs), phosphoinositide 3 kinase (PI-3K), and Akt. Interestingly, soluble CD154 significantly inhibits Fas-mediated apoptosis in T cell leukemia-lymphoma cell lines, Jurkat E6.1 and HUT78 cells, an important hallmark of T cell survival during malignancy progression. These anti-apoptotic effects were mainly mediated by the activation of the PI-3K/Akt pathway but also involved the p38 and the ERK1/2 MAPKs cascades. Our data also demonstrated that the CD154-triggered inhibition of the Fas-mediated cell death response was dependent on a suppression of caspase-8 cleavage, but independent of *de novo* protein synthesis or alterations in Fas expression on cell surface. Together, our results highlight the impact of the CD154/α5β1 interaction in T cell function/survival and identify novel targets for the treatment of malignant disorders, particularly of T cell origin.

## Introduction

CD154, also known as CD40 ligand or gp-39, is a 33 kDa type II transmembrane protein that belongs to the tumor necrosis factor (TNF) superfamily. Although it was initially found on activated CD4-positive T cells, it is now evident that CD154 is expressed on various cells of the immune system [1,2]. The interaction of CD154 with its classical receptor on B cells, CD40, a member of the TNF receptor (TNFR) family, is of critical importance for immunoglobulin isotype switching during humoral immune response [3]. In addition, this axis also plays a predominant role in cell-mediated immunity, through the up-regulation of adhesion and co-stimulatory molecules, and the production of pro-inflammatory cytokines, chemokines, growth factors, matrix metalloproteinases and procoagulants [4,5,6,7]. Because of its implication in the above described responses, CD154 has been linked to multiple inflammatory conditions, to anti-tumorogenic immune functions but also to survival/proliferation of cancer cells [8,9,10,11,12]. Indeed, circulating levels of soluble CD154 (sCD154), which originate from the proteolytic cleavage of membrane-bound CD154 at the surface of activated T cells and platelets, have now emerged as strong indicators of immune activity in inflammatory diseases [13,14,15,16] and of prognosis level in some types of cancers [17,18,19]

Although CD40 represents the classical CD154 receptor, additional binding partners of potential importance in CD154-mediated inflammatory reactions have been described, namely the αIIbβ3 [20], αMβ2 [21] and α5β1 integrins [22]. Each of these receptors interacts with CD154 in a specific manner. While only inactive α5β1 [22] and active αMβ2 [21] bind to CD154, αIIbβ3 [20,23] in both inactive and active forms may bind to CD154. Indeed, distinct residues of CD154 are involved in its binding to CD40, α5β1, and αIIbβ3, while residues required for αMβ2 binding are shared by CD40 [24]. The interaction of CD154 with αIIbβ3 is required for thrombus stabilization [20], while its interaction with αMβ2 may be involved in leukocyte accumulation and neointimal formation during atherogenesis [21]. With respect to the α5β1/CD154 interaction, we reported that binding of CD154 to α5β1 of human monocytic cells induces several signaling events that may modulate cell function [22]. However, the physiological relevance of this interaction remains uncharacterized.

Integrins and particularly the β1 integrins have been shown to inhibit apoptotic events in T cells of normal or malignant nature. Indeed, ligation of β1 integrins on surface of T cell acute lymphoblastic leukemia (T-ALL) cell lines or primary T cells was shown to reduce apoptosis of these cells in response to cell activation [25], to cell starvation [26] or to Fas stimulation [27,28]. Such apoptosis control induced by the engagement of β1 integrins in T-ALL cell lines was shown to involve activation of several signaling cascades such as the Protein-Phosphatase-2A, the MAPK ERK, the focal adhesion kinase, the MAPK p38 leading to reduced caspase activation and/or sustained Bcl-2 anti-apoptotic protein expression [26,27,28]. Interestingly, adhesion-mediated signaling via α4β1, α5β1 and α2β1 protected malignant T cells from doxorubicin-induced cell death response conveying as such resistance to chemotherapy [29,30].

This led us to hypothesize that the interaction of α5β1 integrin with its novel ligand CD154 may represent an important axis in T cell crosstalks and cell resistance to apoptosis, hallmark of T cell malignancies. Here, we show that soluble CD154 binds to the human T-ALL cell line, Jurkat E6.1 in an α5β1-dependent manner. This is associated with the activation of key survival signaling pathways, such as the MAPKs (p38 and ERK1/2) and phosphoinositide 3 kinase (PI-3K)/Akt cascades. More importantly, data presented herein, indicate that CD154 is capable of significantly protecting T-cell leukemia or lymphoma cell lines from Fas-mediated death, through activation mainly of the PI-3k/Akt pathway but also of the p38 and ERK1/2 MAPK cascades as well as via the inhibition of caspase-8 cleavage. This study adds insights into the role of the CD154/α5β1 interaction in T cell function and its resistance to apoptosis-inducing events in many diseases in general and hematopoietic malignancies in particular.

## Results

### Soluble CD154 binds to Jurkat E6.1 T cells in an α5β1-specific manner

**Fig 1:**
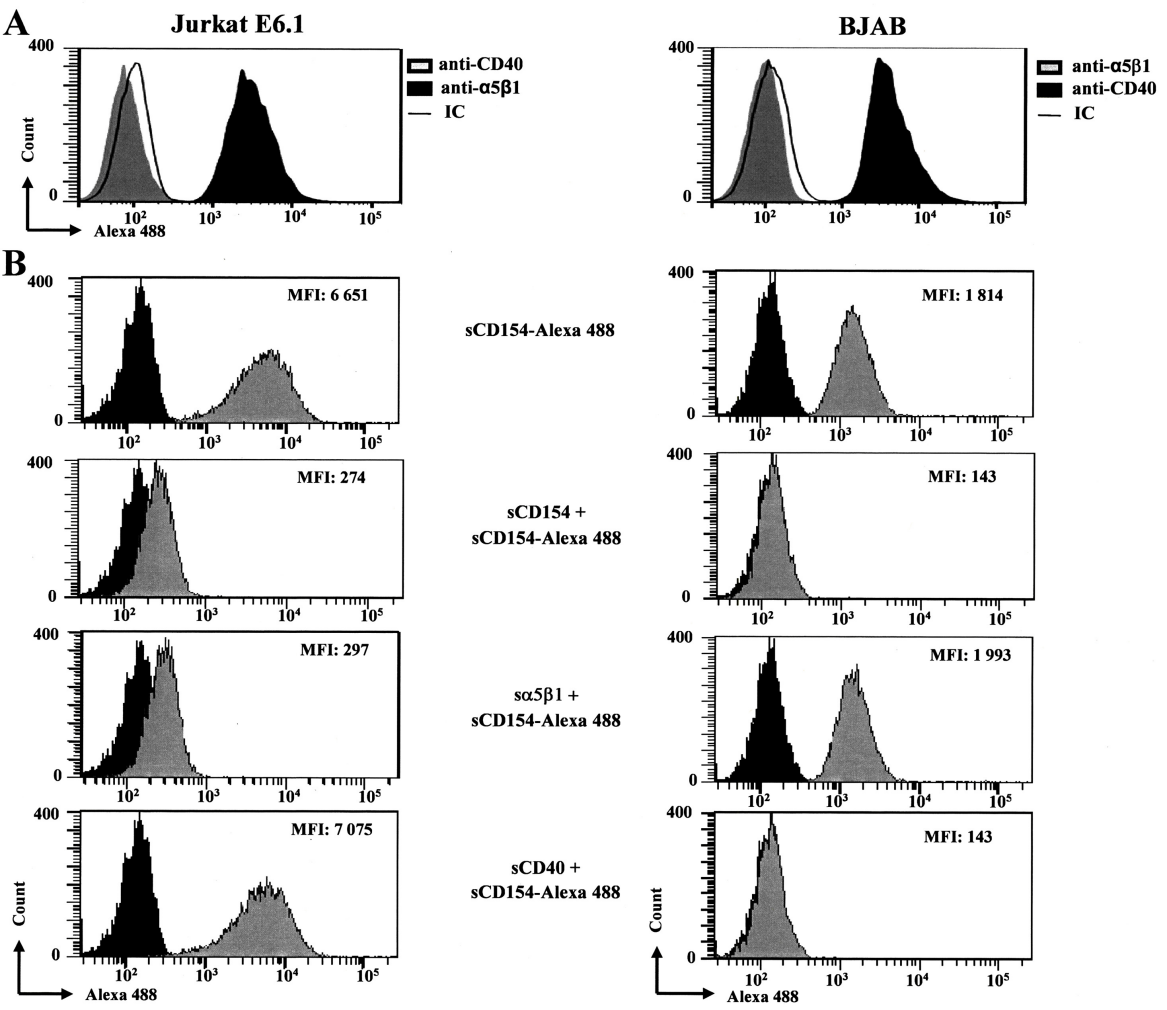
Binding of sCD154 to Jurkat E6.1 T cells is α5β1-dependent. *(A)* Jurkat E6.1 and BJAB cells were incubated with anti-α5β1 JBS5 mAb, anti-CD40 G28.5 mAb, or IgG isotype control (IC) followed by mouse anti-human Ig labeled with Alexa 488. *(B)* Cells were incubated with Avidin-Alexa 488 (negative control, black plots) or sCD154-Alexa 488 (20ng/sample, grey plots) in the absence or presence of 10 fold molar excess of unlabelled sCD154, sα5β1, or sCD40. Expression and binding was then analyzed by flow cytometry. Overlay plots shown are representative of at least 3 independent experiments.

Malignant T cells such as T-cell acute lymphoblastic leukemia (T-ALL) cell lines express the α5β1 integrin [31]. We aim here at investigating the α5β1-dependent binding of CD154 to these cells, using the human T-ALL cell line, Jurkat E6.1 as a model. For this purpose, we have first analyzed the expression of CD154 receptors, CD40, αMβ2, α5β1, and αIIbβ3 on the surface of Jurkat E6.1 cells, by flow cytometry. The Burkitt’s lymphoma B cell line, BJAB was used as a control as it is known to be negative for α5β1 expression [24,32]. Our data show that Jurkat E6.1 T cells express only α5β1, while BJAB cells express only the CD40 receptor (Fig 1A). It is worth mentioning here that CD40 expression was assessed using two anti-CD40 antibodies (Abs), the clone G28.5 (Fig 1A) and the clone 5C3 (data not shown), both of which yielded comparable results. No detectable αIIbβ3, known to be expressed specifically in platelets, or αMβ2, known to be expressed on monocytes, dendritic cells, and polymormorphonuclear cells, was observed in both cell types (data not shown). We then analyzed the binding of sCD154 to these cells using sCD154 labeled with Alexa 488 (sCD154-Alexa). As shown in Fig 1B, both cell lines, Jurkat E6.1 and BJAB cells, exhibited significant binding of sCD154-Alexa. Pre-incubation with 10 fold excess of uncoupled sCD154 strongly reduced and abolished sCD154-Alexa binding to Jurkat E6.1 and BJAB cells respectively, confirming the specificity of our experimental approach. More importantly, co-incubation of sCD154-Alexa with soluble α5β1 completely reversed sCD154 binding to Jurkat E6.1 cells, but not to BJAB cells. Inversely, co-incubation of sCD154-Alexa with soluble CD40 had no effect on sCD154 binding to Jurkat E6.1 cells, while it entirely reversed its binding to BJAB cells. Taken together, these data indicate that sCD154 binds to Jurkat E6.1 T cells in an α5β1-dependent manner.

### Binding of sCD154 to T cells induces intracellular signaling

**Fig 2:**
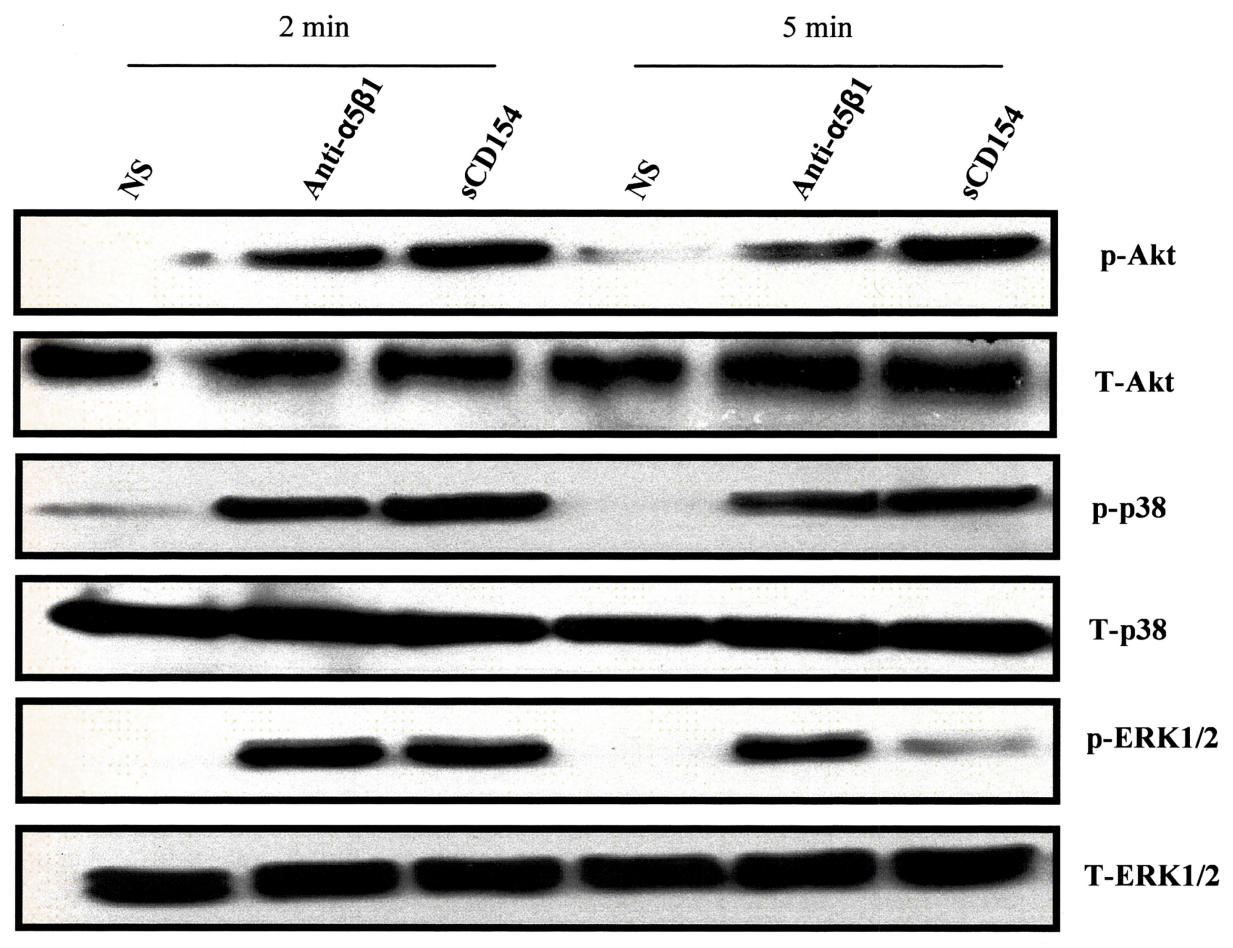
Soluble CD154 induces intracellular signaling in Jurkat E6.1 T cells. Jurkat E6.1 cells were activated with sCD154 (50 ng/sample) or anti-α5β1 JBS5 mAb (500 ng/sample) for the indicated time at 37°C and reactions were terminated by addition of 2X Laemmli sample buffer containing protease and phosphatase inhibitors. Samples were then analyzed by western blot for Akt, p38 and ERK1/2 activation using phospho-specific antibodies. Blots were stripped and reprobed with antibodies against total Akt, total p38 and total ERK1/2. Blots shown are representative of 3 independent experiments.

In order to demonstrate the biological significance of the interaction between CD154 and the α5β1 integrin on T cells, and having confirmed above that α5β1 is the only CD154 receptor expressed in Jurkat E6.1, cells were stimulated with sCD154 and assessed for intracellular events. Interestingly, binding of sCD154 to T cells was associated with the activation of key survival signaling pathways, such as the MAPKs and PI-3K cascades. Indeed, sCD154 induced a significant time-dependent phosphorylation of p38 and ERK1/2, as well as Akt in these cells (Fig 2). In addition, ligation of α5β1 with the agonistic anti-α5β1 antibody, clone JBS5 showed similar activation patterns to those obtained with sCD154, suggesting α5β1 specificity.

### Soluble CD154 inhibits Fas-induced apoptosis in T-cell leukemia or lymphoma cells

**Fig 3:**
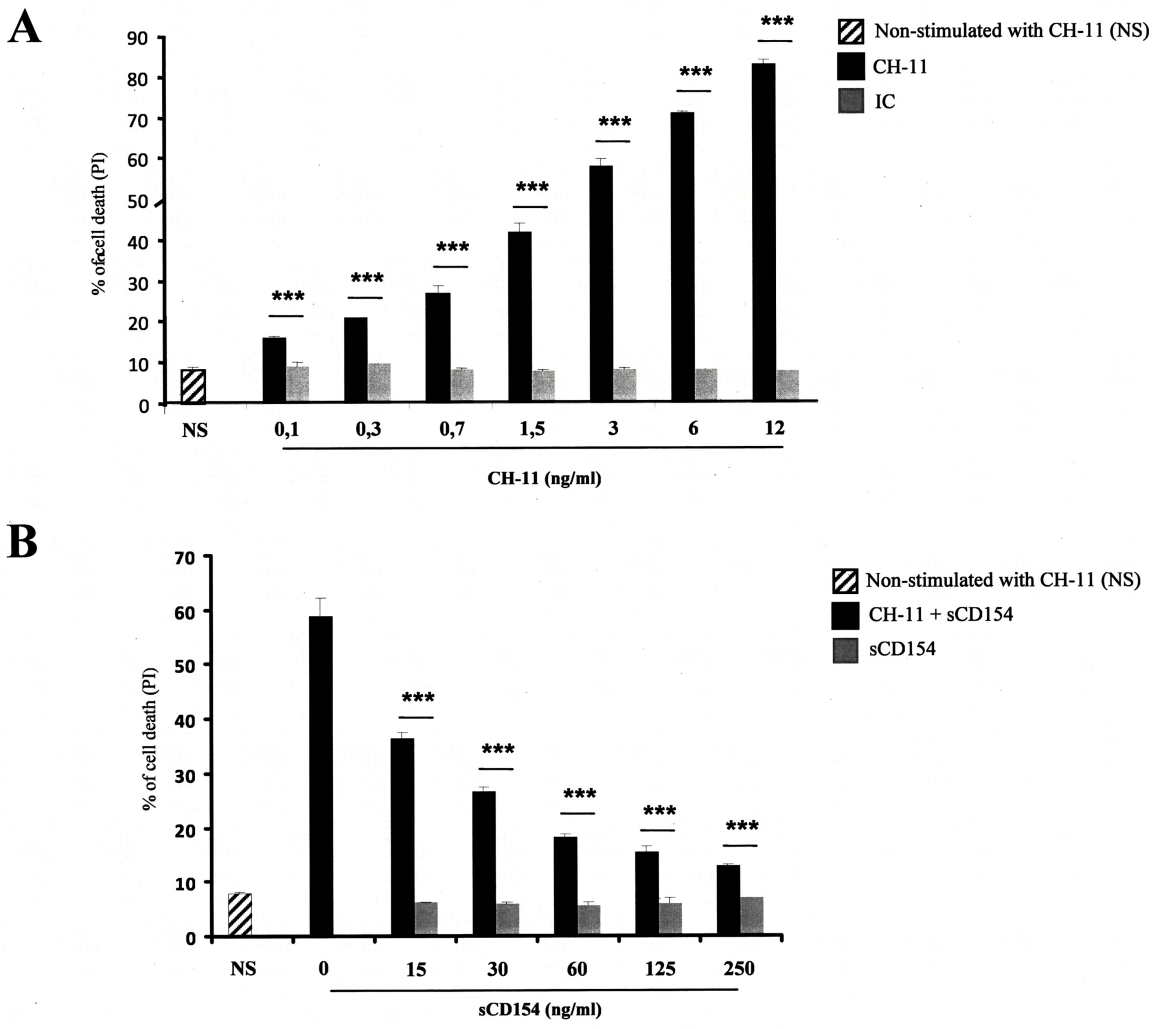
Soluble CD154 inhibits Fas-mediated cell death in Jurkat E6.1 T cells. *(A)* Jurkat E6.1 T cells were left non-stimulated (NS) or stimulated with different concentrations of anti-Fas CH-11 mAb or IgM isotype control (IC) for 18 hours at 37°C and assessed for their cell death response by FACS analysis as the percentage of death obtained by propidium iodide staining. *(B)* Jurkat E6.1 T cells were pre-treated with different concentrations of sCD154, then left non-stimulated (NS) or stimulated with the anti-Fas CH-11 mAb (3ng/ml) for 18 h at 37°C. Cell death was then evaluated as above (± SD, *** p < 0.*001*).

Cell survival and resistance to cell death are fundamental elements of the pathophysiology of malignant diseases [33]. In search of the physiological outcome of the CD154/α5β1 interaction in the function of malignant T cells, and based on earlier observations showing that binding of collagen, an extracellular matrix protein, to some members of the β1 integrin inhibits Fas-induced cell death [27], we evaluated the impact of sCD154 on Fas-induced apoptosis in T-ALL cell lines. For this purpose, Jurkat E6.1 cells were treated with different concentrations of anti-Fas CH-11 mAb and assessed for their cell death response. As expected, treatment with anti-Fas CH-11 mAb induced a significant and concentration-dependent apoptotic effect in Jurkat E6.1 cells (Fig 3A). Interestingly, pre-incubation of cells with sCD154 inhibited Fas-mediated T cell death in a dose-dependent manner (Fig 3B).

**Fig 4:**
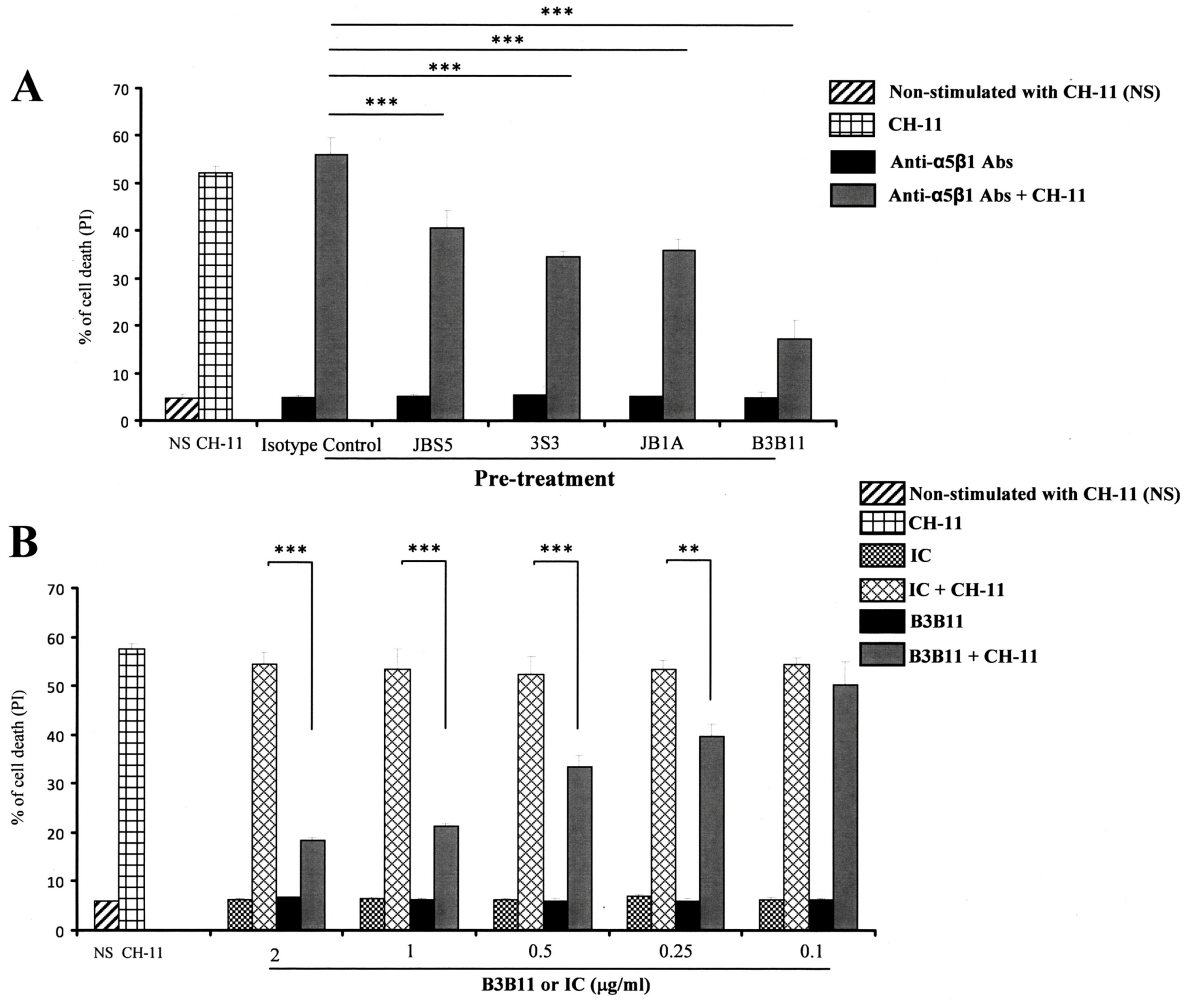
Anti-α5β1 Abs inhibit Fas-mediated cell death in Jurkat E6.1 T cells. *(A)* Jurkat E6.1 T cells were pre-treated with 2 μg/ml of isotype control (IgG, IC) or different anti-α5β1 Abs, JBS5, 3S3, JB1A, B3B11, then left non-stimulated (NS) or stimulated with the anti-Fas CH-11 mAb (3ng/ml) for 18 h at 37°C. Cell death was then evaluated by FACS analysis as the percentage of death obtained by propidium iodide staining. *(B)* Jurkat E6.1 T cells were pre-treated with isotype control (IgG, IC) or different concentrations of the anti-α5β1, B3B11, then left non-stimulated (NS) or stimulated with the anti-Fas CH-11 mAb (3ng/ml) for 18 h at 37°C. Cell death was then evaluated as above (± SD, ** p < 0.*01*, *** p < 0.*001*).

To further confirm the specificity of the α5β1 integrin as the CD154 receptor in play in the resistance of T cells to Fas-induced death, we pre-incubated Jurkat E6.1 cells with different antibodies directed against α5β1, namely clones JBS5, 3S3, JB1A and B3B11, and then treated these cells with anti-Fas CH-11 mAb and evaluated their cell death as above. All anti-α5β1 Abs were capable of inhibiting the Fas-mediated T cell death with the B3B11 clone exhibiting the highest effect (Fig 4A). Indeed, Fig 4B shows the B3B11-concentration-dependent inhibition of the Fas-mediated apoptosis in Jurkat E6.1 cells.

**Fig 5:**
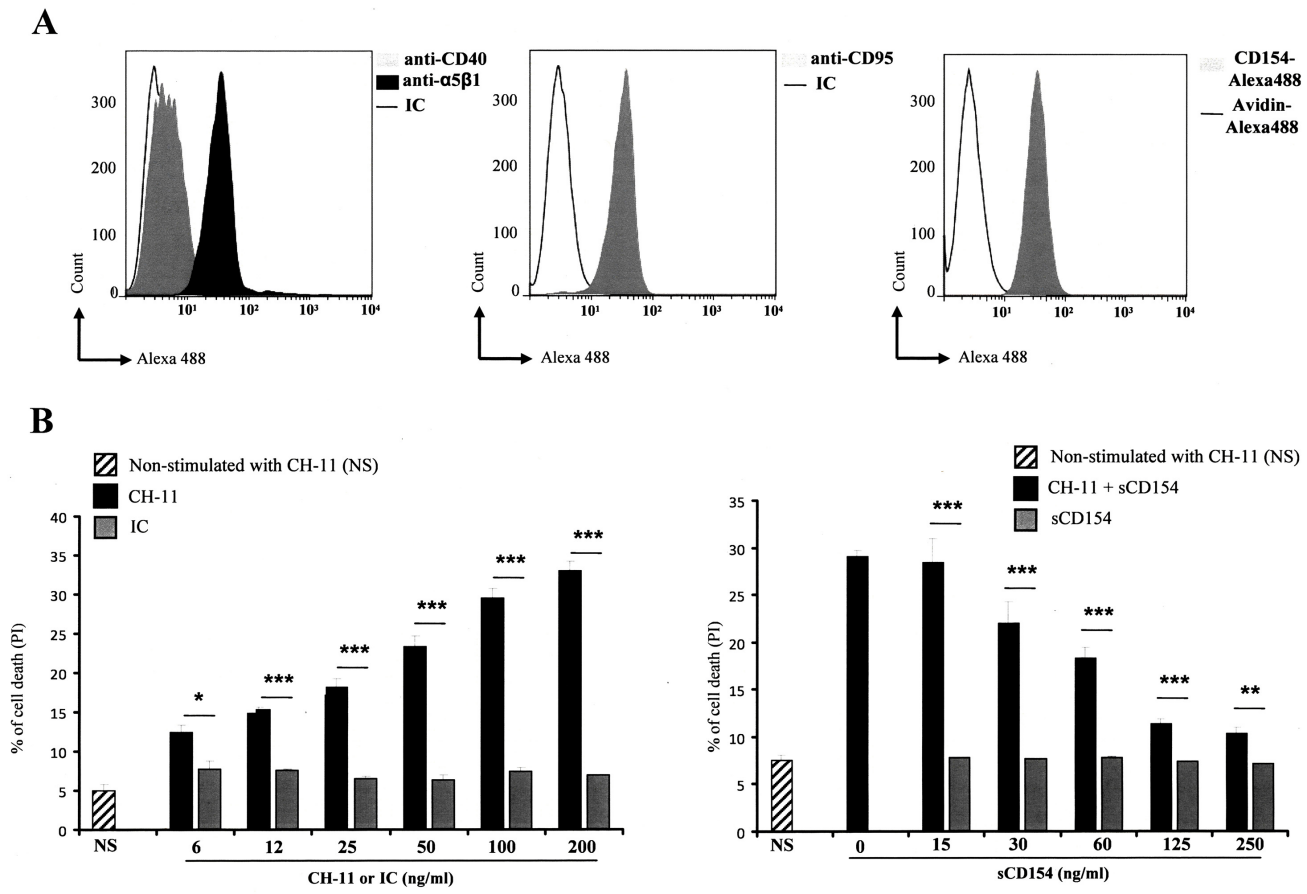
Soluble CD154 inhibits Fas-mediated cell death in HUT78 T cells. *(A*, *Left and middle pannels)* HUT78 cells were incubated with anti-α5β1 JBS5 mAb, anti-CD40 G28.5 mAb, anti-Fas LOB3/17 mAb, or IgG isotype control (IC) followed by mouse anti-human Ig labeled with Alexa 488. *(A*, *Right panel)* Cells were incubated with Avidin-Alexa 488 (negative control) or sCD154-Alexa 488 (20ng/sample). *(B*, *Left panel)* HUT78 T cells were left non-stimulated (NS) or stimulated with different concentrations of anti-Fas CH-11 mAb or IgM isotype control (IC) for 48 hours at 37°C and assessed for their cell death response by FACS analysis as the percentage of death obtained by propidium iodide staining. (*B*, *Right panel)* HUT78 cells were pre-treated with different concentrations of sCD154, then left non-stimulated (NS) or stimulated with the anti-Fas CH-11 mAb (200ng/ml) for 48 h at 37°C. Cell death was then evaluated as above (± SD, * *p < 0*.*05*, ** p < 0.*01*, *** p < 0.*001* CH-11 versus IC).

In order to assess if the CD154-mediated inhibition of Fas-induced cell death is specific to Jurkat cells, or could be also observed in other malignant T cells, we tested this effect of CD154 on Fas-stimulated HUT78 cells, a neoplastic T cell line derived from Sezary syndrome T cell lymphoma [34]. Our data show that HUT78 cells express important levels α5β1 integrin and Fas on their cell surface with no detectable expression of CD40 and exhibit significant binding to sCD154 (Fig 5A). To assess their sensitivity to apoptotic events, HUT78 cells were treated with anti-Fas CH-11 mAb and evaluated for their death response. HUT78 cells, shown to express Fas molecules on cell surface, exhibited significant apoptosis in response to Fas stimulation (Fig 5B). Even though HUT78 cells were less sensitive to Fas-induced cell death than Jurkat cells, their pre-incubation with sCD154 also inhibited this Fas-mediated apoptotic response in a dose-dependent manner (Fig 5B).

Together, these results confirm that signaling events triggered upon the binding of sCD154 to α5β1 of leukemia or lymphoma T cell lines, leads to the inhibition of Fas-induced cell death, a phenomenon that plays a key role in the progression of malignancies, and particularly here of T cell origin.

### Intracellular mechanisms implicated in the CD154-mediated survival of malignant T cells

**Fig 6:**
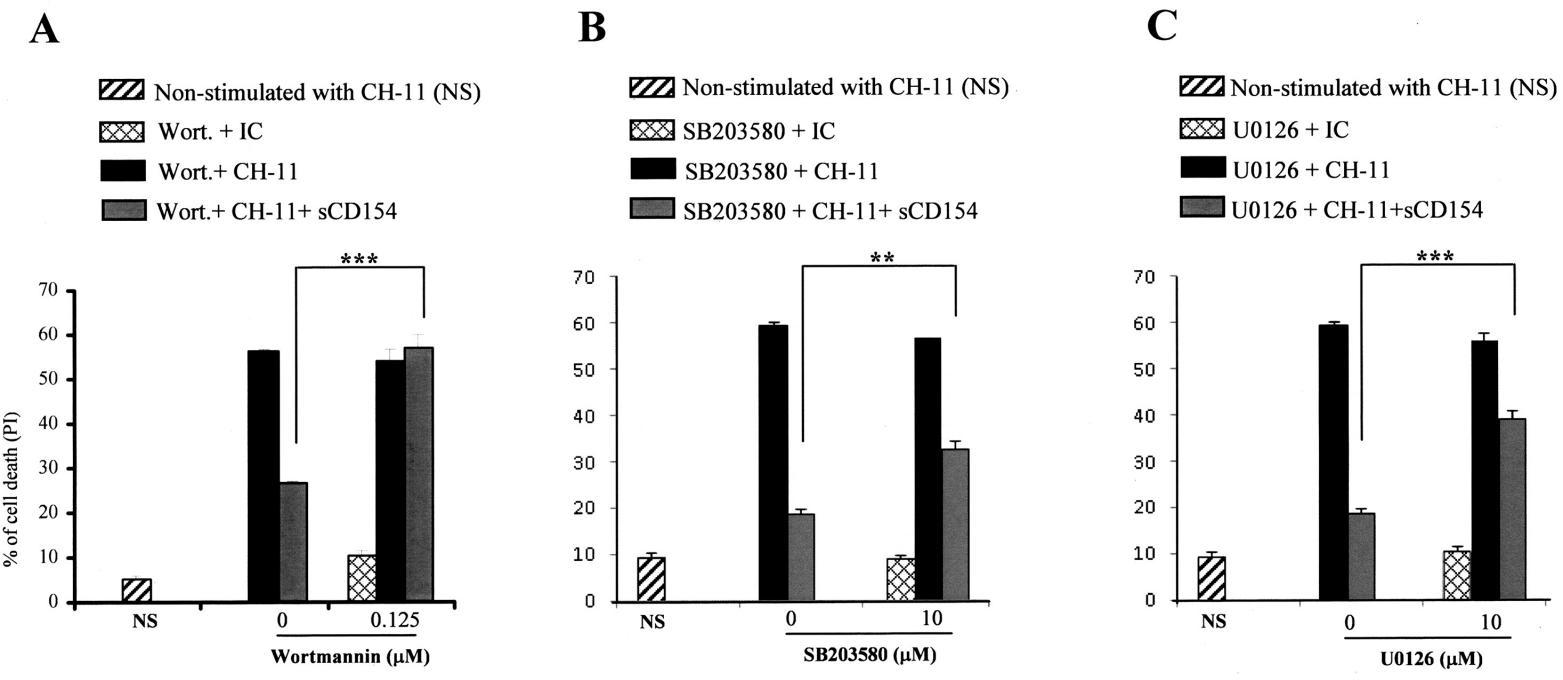
The CD154-mediated inhibition of Fas-induced apoptosis is regulated mainly by the PI-3K/Akt pathway but also by the p38 and the ERK1/2 signaling cascades. Jurkat E6.1cells were pre-incubated with 0.125 μM of Wortmannin *(A)*, with 10μM of SB203580 *(B)* or with 10 μM of U0126 *(C)* for 30 minutes at 37°C. Cells were subsequently incubated with or without sCD154 for an additional 3 hrs, and then stimulated with anti-Fas CH-11 mAb (3ng/ml) or IgM isotype control (IC) for 18 hours at 37°C and assessed for their cell death response by FACS analysis as the percentage of death obtained by propidium iodide staining. Data are representative of means of four independent experiments (± SD, ** *p* < 0.01, *** *p* < 0.001).

To confirm the importance of various intracellular cascades in the CD154-mediated inhibition of Fas-induced apoptosis, specific targeting using biochemical inhibitors, was employed. Jurkat E6.1 T cells were first treated with different concentrations of the PI-3K inhibitor, Wortmannin, the p38 inhibitor, SB203580, or the ERK1/2 inhibitor, U0126 in order to determine the highest concentration of each inhibitor that do not induce a cell death response by itself (data not shown). Following these dose-response experiments, Jurkat E6.1 T cells were treated with sCD154 in the presence of 0.125 μM of Wortmannin, 10μM of SB203580 or 10μM of U0126, then stimulated with Fas and assessed for cell death. As shown in Fig 6A–6C, specific inhibition of the PI-3K/Akt cascade entirely prevents CD154-mediated influence over the Fas-induced apoptosis, while inhibition of the p38 or of the ERK1/2 MAPKs reduced without completely abolishing this CD154-mediated effect. It is worth noting here that another inhibitor of the PI-3K/Akt pathway, the LY294002 also completely abrogated the CD154-mediated survival of Fas-stimulated T cells (data not shown).

Taken together, these results indicate that mainly the PI3K/Akt and to a lesser extent the p38 and ERK1/2 MAPK cascades are mediating the CD154-triggered survival of malignant T cells.

### Soluble CD154-mediated T cell survival does not alter Fas expression and is independent of de novo protein synthesis

**Fig 7:**
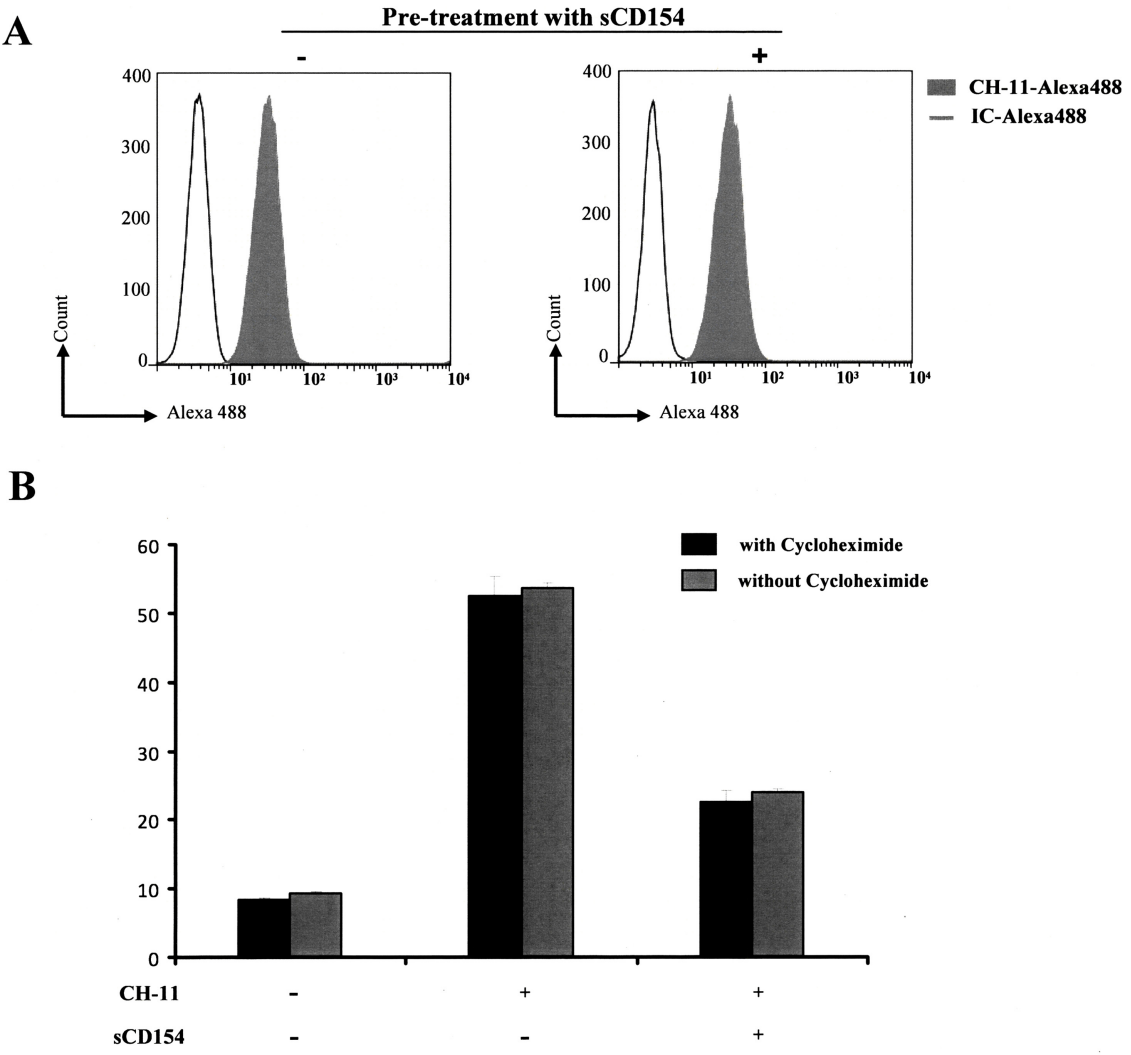
Soluble CD154-mediated inhibition of Fas-induced cell death does not influence Fas expression and is independent of de novo protein synthesis. *(A)* Jurkat E6.1 T cells incubated with medium alone or sCD154 (50 ng/ml) for 3 h were washed and stained with anti-Fas CH-11 labeled with Alexa 488 or IgM isotype control (IC) labeled with Alexa488. Cells were then washed and analyzed by flow cytometry. *(B)* Jurkat E6.1 T cells were pre-incubated with 35 ng/ml cycloheximide or vehicle DMSO for 15 min at 37°C. Samples were subsequently incubated with sCD154 (50 ng/ml) or left untreated for 3 h at 37°C, and cell death was induced by the anti-Fas CH-11 mAb (3 ng/ml) for 18 hours at 37°C. Data are representative of three independent experiments.

Many studies have shown that CD40 activation alters Fas expression in both normal and malignant B cells [35,36,37,38]. Hence, it was important to determine next whether the sCD154/α5β1 interaction affects the level of expression of this critical pro-apoptotic gene. Fig 7A shows that sCD154 protects against cell death though a mechanism that appears independent of Fas membrane expression, given that sCD154 did not alter the level of expression of the receptor on cell surface, assessed by the extent of binding of CH-11 coupled to Alexa488. It is worth noting at this point that since these data are showing that in the presence of an excess sCD154, CH-11 was still capable of binding similarly to its receptor on T cell surface, thus the inhibiting effect of CD154 toward the Fas-mediated cell death response is not due to a competition between CD154 and CH-11 for the Fas-receptor binding. This effect was also independent of *de novo* protein synthesis, as pre-treatment with cycloheximide, an inhibitor of protein biosynthesis, did not reverse sCD154 ability to prevent Fas-mediated T cell death (Fig 7B).

### CD154 inhibits Fas-mediated T cell death by blocking caspase-8 cleavage

**Fig 8:**
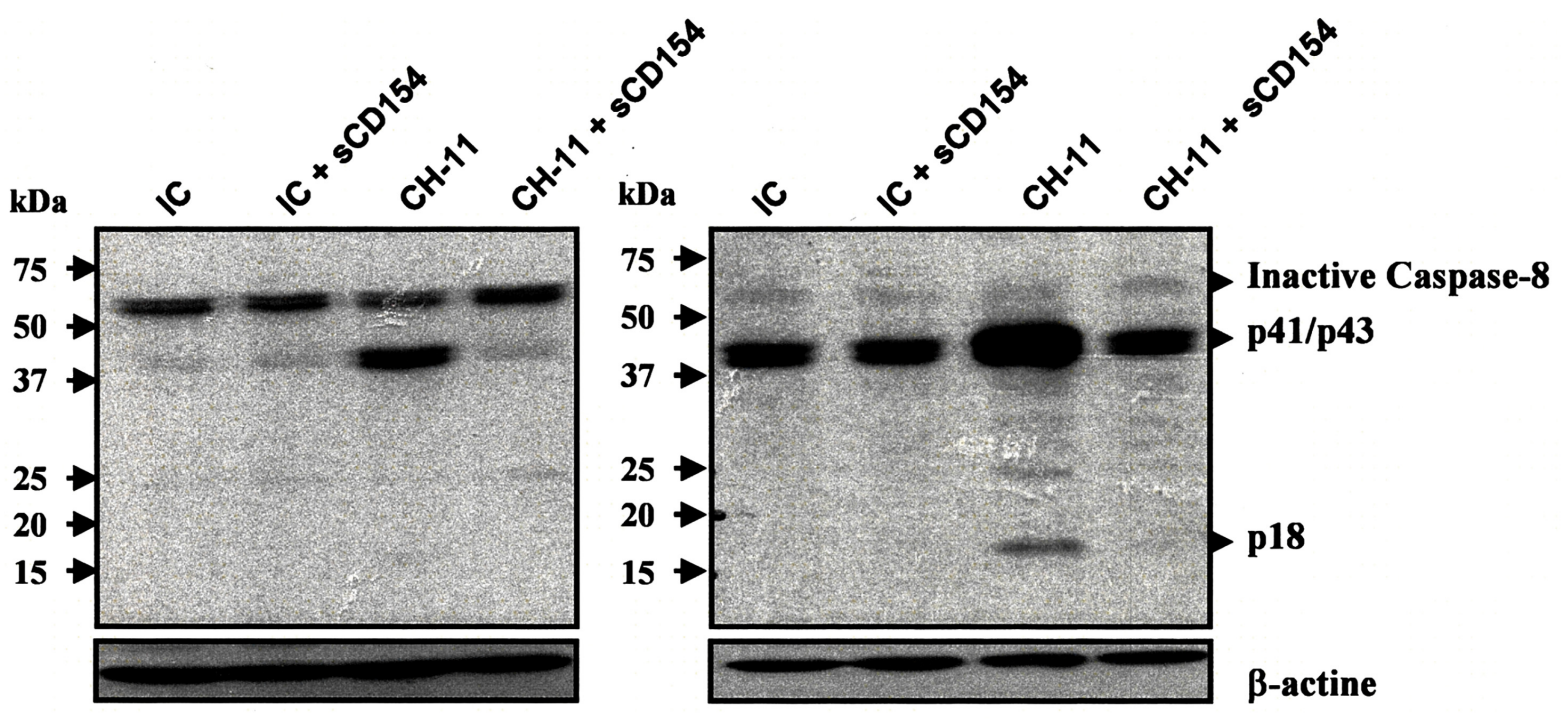
Soluble CD154 inhibits Fas-mediated T cell death by inhibiting caspase-8 cleavage. Jurkat E6.1 T cells were pre-treated or not with sCD154 (50 ng/ml) for 6h at 37°C and stimulated with the anti-Fas CH-11 mAb (12 ng/ml) or with an isotype-matched control (IgM) for 3h at 37°C. Cells were then boiled for 7 minutes and caspase-8 cleavage was assessed by immunoblot using two different anti-caspase-8 antibodies (clone 1C12 for the detection of full length caspase-8 [57 kDa] as well as cleaved intermediates [p44/p43 and p18 fragments] (left panel) and clone 18C8 for the detection of cleaved intermediates only [p44/p43 and p18 fragments]) (right panel). Blots shown are representative of 3 independent experiments.

Caspase-8 cleavage is a critical aspect of Fas-mediated cell death. This ultimately leads to cytochrome c release from the mitochondria and caspase-3 activation [39]. To investigate the role of sCD154 in inhibiting caspase-8 cleavage, two different mAbs against caspase-8 (1C12 mAb for the detection of the full length caspase-8 [57 kDa] as well as cleaved intermediates [p44/p43 and p18 fragments] and 18C8 mAb for the detection of cleaved intermediates only [p44/p43 and p18 fragments]) were used in a Western Blot technique. Fig 8 shows that treatment of Jurkat E6.1 T cells with the pro-apoptotic anti-Fas CH-11 mAb induces caspase-8 cleavage, as compared to non-stimulated cells. However, pre-incubation of cells with sCD154 prior to anti-Fas CH-11 mAb treatment, entirely prevented Fas-mediated caspase-8 cleavage. These data outline the mechanism of CD154-mediated inhibition of apoptosis in T cells and highlight the physiological relevance of the CD154/α5β1 axis in these cells as a promoter of cell survival and resistance to apoptosis.

## Discussion

Multiple lines of evidence now support the implication of CD154 in the development of chronic inflammatory and autoimmune disorders [8,40], as well as malignant disorders [11,12]. Even though most CD154-driven pathways have been attributed to its interaction with CD40, additional binding partners have been described namely the integrins αIIbβ3, αMβ2 and α5β1 [20,21,22]. These receptors add complexity to the diverse interplays in which CD154 takes part. In this study, for the first time, we reveal the importance of the CD154/α5β1 interaction in the function of T lymphocytes of malignant nature. Using T-ALL cell lines, we showed that binding of sCD154 to α5β1 on these cells induces activation of key survival signaling pathways such as the MAPKs (p38 and ERK1/2) and PI3K cascades, in a manner that is independent of integrin conformational change or activation. More importantly, we highlight the pathophysiological importance of this axis in T cell survival and resistance to Fas-mediated cell death through inhibition of caspase-8 cleavage.

Malignant T cells constitutively express many types of integrins including the α5β1 [31]. Given the identification of α5β1 as a novel receptor for CD154, we first assessed the capacity of CD154 to bind to the T-ALL cell line, Jurkat E6.1. Our data demonstrate that sCD154 binds to Jurkat T cells, which are negative for CD40, αMβ2 and αIIbβ3. The α5β1 integrin is however not restricted to T cells, as human monocytic cells [22] and bronchial fibroblasts [41] obtained from asthmatic subjects have been previously shown to interact with CD154 in an α5β1-dependent manner. Hence, CD154/α5β1 crosstalks may take place in various cells of immune, inflammatory or malignant nature, contributing as such to the development of inflammatory diseases as well as malignancies, particularly here hematopoietic ones.

Since sCD154 binds to T cell lines in an α5β1-restricted manner, it was essential to determine whether this interaction could induce intracellular signaling. As CD40 signaling in B cells induces the activation of specific signaling pathways, including the p38 and ERK1/2 MAPKs [42], leading to B cell differentiation and survival, our data showed that α5β1 signaling in response to CD154 could also induce activation of these signaling cascades in T cells. In addition to p38 and ERK1/2, we showed that the CD154/α5β1 interaction leads to Akt activation, another critical pathway of CD154-induced B cell activation and differentiation [42]. In parallel, binding of α5β1 to its classical fibronectin ligand also induces the activation of similar signaling pathways including MAPKs [43,44,45], suggesting that α5β1 signaling in T cells is not specific to CD154 but can be also induced by other ligands. Nevertheless, the ability of sCD154 to induce activation of these signaling pathways in T cells is indicative of a new cellular activation pathway of potential importance in T cell function.

In addition to cell adhesion and migration on the extracellular matrix, integrins are also capable of promoting differentiation and cell growth processes in many cell types of normal or malignant nature [46,47]. Interestingly, one of the β1 integrins, the α4β1 was shown to mediate the interaction of myeloma cells and B malignant cells with the bone marrow stroma leading to cell adhesion, migration and/or survival [48,49]. In T-ALL, another β1 integrin, the α2β1 by interacting with collagen I, was capable of mediating bone marrow stroma-induced survival of malignant T cells [26]. We have previously reported that collagen I, by interacting with β1 integrins on surface of T cell lines or even primary T cells reduced cell death in response to Fas stimulation. In these studies, we have shown that these interactions induced activation of the Protein-Phosphatase-2A and of the downstream MAPK/ERK pathway leading to an abrogated Fas-induced caspase-8 activation [27]. More recently, it was demonstrated that the Jurkat E6.1 T cell line stimulated with collagen I exhibited activation of their MAPK p38 pathway and downregulation of their Fas-induced apoptosis [28]. Such response was shown to be mediated by β1 integrins as overexpressing β1 integrins in T cells enhanced their phosphorylation of p38 and abrogated Fas-induced apoptosis while Abs directed against β1 integrins reversed these responses [28]. Interestingly, our group has also reported that the β1 integrin/collagen interaction was responsible for inhibiting doxorubicin-induced apoptosis in a MAPK/ERK-dependent mechanism, promoting as such a chemo-resistant phenotype [30]. Integrin-associated chemo-resistance was again assessed in another study, whereby the interaction of fibronectin with the α5β1 integrin in a Jurkat derivative cell line (JB4, lacking the α4 expression) decreased cell apoptosis in response to the chemotherapeutic agent, doxorubicin probably by enhancing Akt phosphorylation [29]. Given these data we were highly interested in investigating if the novel interaction of the α5β1 with CD154 could also induce anti-apoptotic and survival signals in malignant T cells using a T-cell leukemia model, Jurkat E6.1 and a T-cell lymphoma one, HUT78. Indeed, and in agreement with the above studies, pre-incubation of these cells with sCD154 completely prevented Fas-mediated T cell death, indicating that CD154 may represent a critical element of T cell persistence and survival during malignant disorders. The CD154/α5β1 interaction may thus correspond to a fundamental axis in the progression of tumors, particularly those of hematopoietic type. Our results could explain the enhanced levels of sCD154 observed in hematological malignancies such as acute lymphoblastic leukemia, chronic lymphocytic leukemia, mantle cell lymphoma and multiple myeloma [50] whereby sCD154 could be contributing to the pathogenesis of the disease by enhancing the survival of malignant cells in an α5β1-dependent manner.

Cell survival is tightly regulated by a balance between anti-apoptotic (survival) factors and pro-apoptotic factors and their intracellular signals. As mentioned above, several intracellular mediators including Protein-Phosphatase-2A, ERK and p38 MAPKs, PI-3K and Akt have been shown to regulate cell survival upon ligation of β1 integrins with their natural matrix-derived ligands [27,28,29,51]. In agreement with these results, our data also show that engagement of α5β1 integrin with CD154 induces activation of p38 and ERK1/2 MAPKs and Akt, as discussed above. Furhermore, inhibiting p38 or ERK1/2 MAPKs reduced the anti-apoptotic effect of CD154 in T cells while inhibiting PI-3K completely reversed this effect, confirming the involvement of the p38/ERK1/2 MAPKs and more importantly of the PI-3K/Akt cascade as mechanisms regulating the CD154/α5β1-mediated survival of malignant T cells. In the same line of evidence, others have demonstrated that the constitutive activation of PI-3K/Akt is associated with the resistance of certain cell types such as rheumatoid arthritis synovial-like synoviocytes to death signals including Fas ligand, TNF and TNF-related apoptosis-inducing ligand (TRAIL) [52]. As to the apoptotic signals inside the cell, it is well established that caspase activation and in particular caspase-8 cleavage are key signaling elements of Fas-mediated cell death [39]. Caspase-8 indirectly mediates the release of cytochrome c from the mitochondria and the activation of caspase-3, both of which leads to cleavage of cellular components and programmed cell death. Here, we were able to show that sCD154 by interacting with its receptor on T cells, the α5β1 integrin, inhibits Fas-mediated cell death through inhibition of caspase-8 cleavage, a process that appears independent of *de novo* proteins synthesis. Blocking caspase-8 cleavage was also the underlying mechanism by which a collagen/β1 integrin dyad could reduce Fas-mediated apoptosis in T cell lines [27]. Interestingly, our data herein are further establishing a link between activation of PI-3K/Akt pathway and the inhibition of caspase-8 cleavage.

In conclusion, this study adds significant insights into the role of the CD154/α5β1 interaction in the function of T cells and particularly malignant ones, and shows that this axis promotes T cell survival and resistance to apoptosis. This novel pathway may drive progression of malignant diseases and underlie mechanisms by which malignant cells can escape immune checkpoints or even chemotherapy. Specific inhibition of this dyad may represent a potential therapeutic target in the treatment of T-cell derived malignancies.

## Materials and Methods

### Reagents and antibodies

Recombinant soluble CD154 (sCD154) and recombinant soluble CD40-Fc were generated as previously described [53,54]. Anti-caspase-8 (clone 1C12 and 18C8), anti-phospho-p38, anti-p38, anti-phospho-ERK1/2, anti-ERK1/2, anti-phospo-AKT and anti-AKT antibodies were all procured from Cell Signaling Technology (Beverly, MA). Anti-CD95 or anti-Fas (clone CH-11, an IgM) and anti-actin (clone C4) antibodies came from Chemicon (Temecula, CA). Anti-CD95 (LOB3/17, an IgG) was purchased from Bio-Rad AbD Serotec Inc. (Raleigh, NC). Anti-αMβ2 antibodies came from BD Biosciences (Mississauga, ON) while anti-α5β1 (clone JBS5) and anti-αIIbβ3 antibodies were purchased from Santa Cruz Biotechnology (Santa Cruz, CA). The anti-α5β1 Abs (clones 3S3, JB1A, B3B11) and the soluble α5β1 (sα5β1) were a generous gift from Dr. John A. Wilkins (Manitoba Centre for Proteomics and Rheumatic Diseases, University of Manitoba, Winnipeg, MB). The anti-CD40 mAb (clone 5C3) was purchased from BioLegend (San Diego, CA), while the anti-CD40 antibody (clone G28.5) was obtained from ATCC (Manassas, VA). The Alexa Fluor-488 labeling kit came from Molecular Probes (Eugene, OR), and labeling of sCD154 (sCD154-Alexa488), CH-11 (anti-Fas, CH-11-Alexa488) and Avidin (Avidin-Alexa488) were performed according to the manufacturer’s instructions. The PI-3K inhibitors, Wortmannin and LY294002 were purchased from Calbiochem (San Diego, CA). The p38 inhibitor, SB203580, and the ERK1/2 inhibitor, U0126 were also obtained from Calbiochem. The protein biosynthesis inhibitor cycloheximide was from Sigma-Aldrich (Oaskville, ON).

### Cell lines

The human Jurkat E6.1 and HUT78 T cell lines (ATCC; Manassas, VA) were maintained at 37°C in RPMI 1640 media supplemented with fetal bovine serum (FBS) (5% in the case of Jurkat cells and 10% in the case of HUT cells) and 1% L-glutamine, penicillin and streptomycin.

### Flow cytometry analysis

The cell surface expression of CD40, α5β1, αMβ2 and αIIbβ3 was determined by FACS analysis, as previously described [22]. Briefly, cells were stained with the appropriate control isotype IgGs or respective antibodies (anti-CD40 G28.5 mAb, anti- α5β1 JBS5 mAb, anti-αMβ2 mAb or anti-αIIbβ3 mAb followed by goat anti-mouse IgG-Alexa488 staining. Binding of sCD154 was performed using recombinant sCD154-Alexa488 or with unlabeled sCD154, followed by anti-V5 streptavidin-PE. For competition binding, sCD154-Alexa488 was incubated with sα5β1 or sCD40 (10 fold molar excess) for 1 hour at 37°C prior to binding. Cells were also incubated with unlabeled sCD154 for 1 hour at 37°C prior to addition of sCD154-Alexa488 for fluorescence specificity. All samples were analysed on a BD LSRII flow cytometer (BD Biosciences, Mountain View, CA) and cells were gated by their characteristic forward and side scatter properties.

### Cell stimulation for assessing intracellular signaling

Jurkat E6.1 T cells were incubated for 3 hours at 37°C in serum-free advanced RPMI media 1640 and stimulated with sCD154 or anti-α5β1 JBS5 for the indicated periods of time. Reactions were terminated by addition of 2X Laemmli sample buffer containing protease and phosphatase inhibitors (Sigma-Aldrich, Oaskville, ON). Samples were then boiled for 5 minutes at 95°C and analysed by immunoblot as described below.

### Cell death

Jurkat E6.1 cells were first pre-incubated with sCD154 or vehicle for 3 hours at 37°C prior to treatment with anti-Fas mAb (CH-11) or isotype-matched control (IgM) for 18 hours at 37°C. HUT78 cells were pre-incubated with sCD154 or vehicle for 3 hours at 37°C and then treated with anti-Fas mAb (CH-11) or isotype-matched control (IgM) for 48 hours at 37°C. Cell death was evaluated by FACS analysis as the percentage of death obtained by propidium iodide (Invitrogen, Burlington, ON) staining.

### Caspase-8 activation

Jurkat E6.1 T cells were pre-treated or not with sCD154 for 6 hours at 37°C and stimulated with the anti-Fas clone CH-11 antibody or isotype-matched control (IgM) for 3h at 37°C. Cells were then boiled for 7 min and caspase-8 cleavage was assessed by immunoblot as described below.

### SDS-PAGE and Immunoblot analysis

Proteins were resolved in 8% to 12% sodium dodecyl sulfate-polyacrylamide gel electrophoresis (SDS-PAGE) gels and transferred to nitrocellulose membranes. The membranes were blocked with 5% bovine serum albumin for 1 hour, washed three times with TBS/T (150 mM NaCl, 20 mM Tris, pH 7.4, 0.1% Tween-20) and incubated with the appropriate primary antibody overnight at 4°C. Following washing steps, membranes were labeled with horseradish peroxidase-conjugated secondary antibody for 1 hour, washed and bound peroxidase activity was detected by enhanced chemiluminescence (PerkinElmer Life Sciences, Waltham, MA). Membranes were stripped using the re-blot solution (Millipore, Temecula, CA) and re-incubated with antibodies against total ERK1/2, p38 and Akt.
